# Milk: A Scientific Model for Diet and Health Research in the 21st Century

**DOI:** 10.3389/fnut.2022.922907

**Published:** 2022-06-10

**Authors:** J. Bruce German, Carlito Lebrilla, David A. Mills

**Affiliations:** ^1^University of California, Davis, Davis, CA, United States; ^2^Department of Food Science and Technology, Davis, CA, United States; ^3^Foods for Health Institute, Davis, CA, United States; ^4^Department of Chemistry, Davis, CA, United States

**Keywords:** milk, lactation, genomics, oligosaccharides, bifidobacteria

## Abstract

The origin of lactation and the composition, structures and functions of milk's biopolymers highlight the Darwinian pressure on lactation as a complete, nourishing and protective diet. Lactation, under the driving pressure to be a sustainable bioreactor, was under selection pressure of its biopolymers with diverse functions acting from the mammary gland through the digestive system of the infant. For example, milk is extensively glycosylated and the glycan structures and their functions are now emerging. Milk contains free oligosaccharides; complex polymers of sugars whose stereospecific linkages are not matched by glycosidic enzymes within the mammalian infant gut. These glycan polymers reach the lower intestine undigested. In this microbe-rich environment, bacteria compete to release and ferment the sugars *via* different hydrolytic strategies. One specific type of bacteria, *Bifidobacterium longum* subsp. *infantis*, (*B. infantis*) is uniquely equipped with a repertoire of genes encoding enzymes capable of taking up, hydrolyzing and metabolizing the complex glycans of human milk. This combination of a distinct food supply and unique genetic capability shapes the composition and metabolic products of the entire microbial community within the lower intestine of breast fed infants. The intestinal microbiome dominated by *B. infantis*, shields the infant from the growth of gram negative enteropathogens and their endotoxins as a clear health benefit. The world is facing unprecedented challenges to produce a food supply that is both nourishing, safe and sustainable. Scientists need to guide the future of agriculture and food in response to these 21st century challenges. Lactation provides an inspiring model of what that future research strategy could be.

## Introduction

The world is facing an urgent challenge: transform the existing agriculture and food enterprise into a sustainable, nourishing and health-promoting system. A daunting problem is the lack of knowledge of what we should eat. While necessary, obtaining all of the essential nutrients is not sufficient to health. The tools are emerging to measure diet as a complex ensemble of biomolecules at specific concentrations (1, 2). What is needed, in addition to compositional data, is to determine which and how much of those hundreds of thousands of components should each individual human eat, according to their genotypic variations, phenotypic diversity, life stage and lifestyle? Databases of food composition (Periodic Table of Foods), annotated for bioactivities are emerging as the knowledge resources needed to take advantage of computational biology (3).

As the life sciences advance with powerful new tools of biology and genomics, of big data and artificial intelligence, we are faced with many challenges, from demographics to emerging pathogens. Ideally, a goal of health is prevention. The aim is to understand biology: how to intervene pro-actively, build individual defenses and protections that prevent the development of disease. Prevention is challenging. Interventions must act on healthy individuals. There is not the simplifying focus of disease diagnostics, there is no disease to diagnose. What targets improve performance, while protecting and preventing diseases in healthy individuals? The cost-benefit ratio is different. If one is suffering from a disease, then the costs of reversing that disease are tangible, quantifiable and specific. The risk of side effects of disease therapeutics can be evaluated within a context. What costs are justified to prevent a disease that one is never going to get? Even more profoundly, an intervention that lowers the risk of one disease but increases the risk of another is a hollow prevention. What scientific strategy would allow investigators to understand how to improve the health of healthy individuals, to act on preventing all diseases and to do so without putting any individuals at risk?

The biological history of mammalian lactation is a process of evolutionary selection of the output of that tissue: milk, sculpted by infant survival and long term genetic success. Mammalian mothers literally dissolve themselves to make a complete and comprehensive diet for their infants. From the earliest pre-mammals, secreting fluids from a hyperactive sweat gland (4), generation after generation, selective pressure rewarded mothers whose lactation secretions gave their offspring a competitive advantage *via* diet, milk. The combination of cost to the mother and advantage to the infant has yielded the rosetta stone for scientific discovery of nourishment, and of the entire principle of diet. Milk nourishes healthy infants, guides their development, protects them from biological and chemical threats and equips them for the complex environments that they will face, life long. This article describes the implementation of a research strategy based around lactation as a scientific focus.

**Figure 1:**
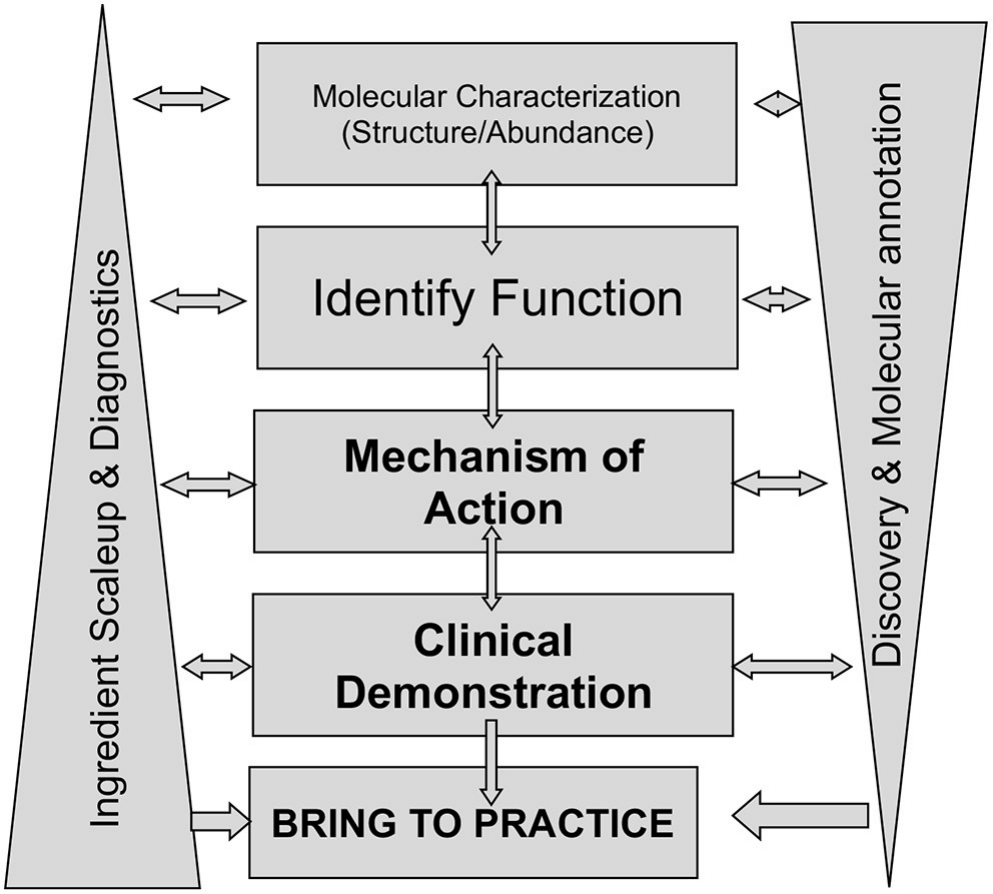
Strategic model for research and development of lactation and its biological product milk.

A basic strategy to study milk is summarized in Figure 1. The goals are to build a map, molecule by molecule, target by target, of how milk achieves its benefits to health. A range of disciplines must work in open collaboration of parallel discovery and innovation. One aim is to identify the components of milk, their structures, abundances and variation within and across mammalian lactation. Another aim is to develop methodologies to isolate these components in purity to enable detailed mechanistic investigations. Another aim is to use a range of biological models in the presence and absence of those isolated components. Once mechanisms of action are discovered, they must move into clinical tests of efficacy. This aim requires insights into the utility of discovered mechanisms: what is the breadth of efficacy across lifespan and lifestage; what diagnostics identify need among the population and what diagnostics provide absolute markers of efficacy. The final imperative is to bring the discoveries to practice as innovations for human benefits.

## Toolsets

### Lactation Genomics

The tools to understand lactation are from genomics to physiology. Whole genome sequencing provides the basic knowledge set. The challenge is to identify and annotate genes associated with lactation. The goals for lactation genomics were propelled by a diverse group of scientists participating with the International Milk Genomics Consortium (5). The diversity of lactation across mammalia is an important asset, so the goal of the IMGC was to assemble a variety of entire genomes from marsupials to humans (6). Comparing these genomes formed the basis for interpreting the evolution of mammalian lactation (7), the expression of genes during lactation (8), the sequences (9) and digestibility of proteins (10), the sequences of peptides (11) and their formation (12), the biosynthetic pathways of glycans (13), the variation among women due to genetic diversity (14), timing of lactation (15), diet (16) and the biosynthesis of lipids (17).

### Lactation Analytics

Milk is a challenge for analytical chemistry. From small molecules to entire cells, milk is a cornucopia of biomolecules varying in size and concentration by orders of magnitude. All the essential nutrients are in milk, each present within a matrix that enhances their bioavailability and controls their chemical reactivity. These matrices that enhance bioavailability impede the characterization of the molecules analytically. Every class of biopolymer is present in milk, all the substrates and intermediates in their synthesis. Milk oligosaccharides were typical. Entire methodological platforms had to be developed for this one biopolymer class alone (18). Oligosaccharide method development required innovative approaches to initial separation, liquid chromatography on novel stationary matrices, mass spectrometry techniques including highly sensitive time of flight and triple quadropole mass spectrometry. The construction of structurally annotated databases of mass spectra was necessary to automate high throughput (19).

### Lactation Bioseparations

The scientific investigations to discover biological actions of milk require that the components be available as purified research materials in quantities and purities maintained in their native conformations for the multiple assays by which investigators address their hypotheses. Also, human milk is a rare and valuable material and simply accessing milk as research material is and should be a process of regulatory, safety and ethical formalities. Combinations of traditional separation technologies and bioguided separations were needed (20). Strategies such as physical separation of milk components by size achieved enrichment in oligosaccharides but retained contamination by peptides and lactose (21). Peptides are an important biological resource in milk but vary widely in abundances and structures depending on the stages of lactation, treatment of milk etc. (22).

## Targets of Biological Function

The evolutionary history of mammalian lactation is remarkable across all of biology. Once begun, the complex interplay between the composition of epithelial secretions and the success of offspring set in motion a Darwinian engine of diet for protection and nourishment of the mother-infant dyad (23). The challenge of annotating lactation is in identifying their mechanisms of function. The challenge of milk research is to understand their role within infants (24). Yet, what are possible actions that would lead to a selective advantage in the mother-infant dyad? Complex oligosaccharides provide an example.

### Milk Oligosaccharides and the Perplexing Lack of Digestion

Glycans are abundant across the tree of life and the most abundant biopolymer in the biosphere (25). Despite their importance they are not sequence encoded but products of enzymatic metabolism. The enzyme specificity to produce glycan structures limits the number of biological structures that are found relative to the enormous number of structures that are mathematically possible. This difference between biologically feasible and mathematically possible has led to the concept of bio-defined analytics (C. Lebrilla, 2000, unpublished). Combining biology with chemical analysis has guided analytical strategies to catalog the glycan structures present in milk and a variety of organisms (26). Structures of glycans include monosaccharide composition, branching, the stereospecific linkages of those sugars all leading to multiple isomers even for a single net atomic mass. Glycan structures are both free and bonded to proteins, peptides or lipids again by enzymatic synthesis. Every glycan, in each sample, must be explicitly analyzed to be identified (14).

The oligosaccharides of human milk have been attractive to scientists because they are free, abundant (1-2% w/v) and yet indigestible by the neonate. They are perplexing to annotate: why would mothers “dissolve themselves” to produce these biopolymers in such abundance? The scientific challenges posed by this apparent paradox propelled laboratories to pursue the analytical platforms to identify and annotate them (27).

### The Bacterial Support Functions of Human Milk Glycans

The structures of milk oligosaccharides have been selected, in part, for an unusual biological value: NOT to be consumed by infants. Research on oligosaccharides in human milk has established as one function, that they support the growth of specific bacteria notably strains of the genus *Bifidobacterium* (28). While the mechanisms and extent of microbial diversity in breastfed infants are still being actively documented, the basic observation that bifidobacterial species dominate the microbiota of breastfed infants around the world compared with formula-fed infants has been well-established (29). How an intestinal microbial ecosystem maintains a dominant and consistent bacterial population in the face of repeated and diverse inoculations with environmental microorganisms has been largely speculative until recently. The idea launched by Gyorgi that oligosaccharides were a Bifidus factor (30) was unfortunately insufficiently specific. Oligosaccharides do not stimulate the growth of the entire genus of *Bifidobacterium* in general. *Bifidobacterium* represent a broad genera of bacteria whose members occupy a wide range of ecological niches. Though first identified microscopicslly by Tissier in the 19th century in breast fed infants only recently has research recognized the unusual specificity of the strains of *Bifidobacterium* that dominate the intestinal microbiome of breast fed infants (31–33). Intensive studies revealed the remarkable interaction between the stereospecific linkages of milk oligosaccharides and the genetic repertoire of glycosidases and solute binding proteins that provide these bacteria a distinct competitive growth advantage within the intestine of the breast fed infant (34).

### Bifidobacteria and the Colonization of the Infant Microbiome

The colonization of the infant by microorganisms begins at birth (35). The consensus of microbiome research argues that the infant gut is ostensibly sterile at birth and those organisms that may have arrived into the amniotic compartment prior to delivery are not competitive once the “flood” of exogenous microorganisms (bacteria, yeast, fungi, viruses) that accompany a normal human birth. These initial inocula are the first of a continuous wave of inoculations of the infant from the environment (36). The mode of delivery, vaginal or by C-section has been noted to alter the gut microbiota of term infants in early life (37), however, these observations are mainly of infants within a restricted microbial environment, the modern hospital delivery room. Infants delivered vaginally acquire bacterial communities resembling those of maternal vagina and fecal microbiomes, while C section babies initially reflect a microbiota resembling that of maternal skin. Infants delivered vaginally exhibited higher abundances of Bacteroidaceae and lower abundances of Enterococcaceae, Pasteurellaceae, Carnobacteriaceae, and Gemellaceae compared to C section delivered infants (38). Knowing that each infant is inoculated with a diverse array of organisms, a goal was to understand the role of that environment and milk components simultaneously in guiding the distinct microbiological community in the breast fed infant. Which microorganisms utilize and grow on specific components of milk (39)? Many components from milk, in isolation, can support microbial growth. Thus, enabling technologies were needed: isolating potential growth substrates in pure form from milk and media for bacterial culture assays that include all of the nutrient requirements for growth, but lack a carbon fuel source. Into these media can then be added the components of milk that are expected to arrive at different sections of the intestine (39). The complex oligosaccharides from milk were isolated to assess bacterial growth on those undigestible components of milk that arrive at the lower intestine. Surprisingly, initial growth experiments did not observe significant growth of bacteria when human milk oligosaccharides were the sole source of carbon in the otherwise supportive medium (40). Among gut-related bacteria tested (including *Lactobacillus, Clostridium, Eubacterium, E. coli, Veillonella, Enterococcus* isolates) only *Bifidobacterium* and *Bacteriodes* species grew to high cell densities yet, growth was strain specific (41). Robust growth on HMO was found just in a select group of *B. bifidum* and *B. longum* subsp. *infantis* (*B. infantis*) strains. In these same growth conditions even isolates of *B. longum* subsp. *longum* and *B. breve* showed poor growth and strains of *B. adolescentis*, and *B. animales* were unable to grow on HMO (41).

Any ecosystem is driven by accessible food. The lower intestine of the breast fed infant is supplied by those components of milk that are not digested nor absorbed by the infant in the upper intestine. Thus, those bacteria capable of accessing oligosaccharides are provided a competitive advantage by the infant's mother's milk. Nonetheless, only the combination of microorganisms growing on the oligosaccharides coded by lactation genes from each infant's mother that confer a selective advantage to infant success would be rewarded through evolution. The outcomes of that Darwinian engine, pathogen protection to immune education are continuing to emerge as novel mechanisms of *Bifidobacterium* dominated microbiome actions (42).

One defining set of traits for colonic bacteria is their ability to degrade biopolymers and access the monomeric sugars, amino acids etc., in that environment. How they do that is important. Most intestinal bacteria secrete extracellular glycosidase enzymes into the luminal environment and these enzymes catalyze the hydrolysis of complex glycans and liberate free sugars extracellularly. Free sugars are taken up by bacteria and metabolized. Select strains of bifidobacteria use extracellular lacto-N-biosidase activity to break down oligosaccharides (43). Some bacterial strains, notably *B. infantis*, pursue a different strategy of transporting oligomeric structures into the interior of the cell and breakdown reactions occur internally. This internal feeding strategy confers an advantage to the host by blocking the liberation of simple sugars into the lumen that other organisms can utilize. Cross feeding liberated sugars to other organisms is a known mechanism to promote the growth of undesirable, opportunistic pathogens (44).

The discovery that growth of bacteria on milk oligosaccharides was a strain specific, gene driven process and that *B. infantis* ATCC15697 was uniquely capable phenotypically, prompted the goal to sequence its genome and begin the process of annotating its unique capabilities. One of the joys of being a scientist is those occasions when you are witness to the sheer elegance of biology. The genetic repertoire of *B. infantis*, was one of those rare moments in which scientific discovery revealed that elegance (31). This specific strain provides the field of microbiome research with insights into the traits associated with capabilities to thrive within the anaerobic intestine including genes providing the strain its phenotype (31). Breast fed infants that are exposed to such HMO consuming strains are colonized by them and in turn achieve direct and indirect benefits. Those benefits even include the protection from the horizontal transfer of virulence and antibiotic resistance traits (45). These benefits are consistent with the concept that the oligosaccharides produced by the mammary gland and the emergence of oligosaccharide consumption gene clusters in specific strains of bifidobacterial strains are an example of symbiotic co-evolution.

The principle of nourishment as the center of cross-kingdom partnerships is not unique to lactation. Glycan based nourishment appears to be at the center of most cross-kingdom symbioses from plants feeding pollinating insects with sugar rich nectar (46) to roots feeding nitrogen fixing bacteria (47). This same strategy emerging from evolution of milk feeding a metabolically distinct and mutually beneficial bacterial population (mutualism) in infants is another example. The challenge is what do we learn by understanding it?

Evidence from epidemiology, mechanistic insights and increasingly prospective interventions shows that the mutualism between human breast milk and the *B. infantis* commensal is important, yet fragile. The importance was first suggested by premature infants. Infants born premature, by Cesarian section, are placed in an incubator. At that point the immediate hospital environment serves as the inoculating reservoir of seeding microorganisms. In such an environment, the explicit steps taken to prevent cross-patient pathogen transfer, (scrupulous hygiene, sanitation, etc.) have the unintended consequence of preventing the transfer of commensal organisms as well. The first indications of the outcomes of that environment emerged in studies comparing the explicit inoculation of candidate organisms. Studies used *in-vivo* administration of *B. infantis* to premature infants fed either formula or breast milk. Breast milk-fed infants, when supplemented with *B. infantis* saw increases in fecal bifidobacteria and decreases in γ*-Proteobacteria* compared with a formula-fed group (48).

Following on those initial studies, *B. infantis*, used clinically, has already been demonstrated to significantly impact the development of inflammation (49), autoimmunity (50) and necrotizing enterocolitis and mortality of premature infants (51). Thus, understanding how mothers are shaping the protective milk-oriented microbiota (MOM) of their infants through breast milk is an urgent model for guiding microbial communities at all ages.

## Conclusions

The deconstruction of human milk through a highly interactive and multi-disciplinary program of research has illuminated the profound interactions between mammals and their resident bacteria. The traditional view of bacteria on and in humans is that they are potentially pathogenic and deleterious. While some bacteria are unquestionably deleterious to animal health, this simple concept that all bacteria are deleterious is incompatible with the realization that human breast milk contains abundant undigestible matter that explicitly feeds a specific strain of *B. infantis*. Research must now pursue studies that illuminate all the reasons why selective pressures through evolution have favored this remarkable partnership.

## Author Contributions

The authors jointly wrote and edited the manuscript. All authors contributed to the article and approved the submitted version.

## Funding

This work was supported by Peter J Shields Endowed Chair (DAM) University of California, Davis, NIH (HD059127, R21AT006180, R01AT007079, R01AT008759, R01HD061923, R01HD065122, and U01CA179582) and Training postdoc (F32HD093185, F32AT006642, and F32AT008533).

## Conflict of Interest

The authors co-founded the company Evolve Biosystems.

## Publisher's Note

All claims expressed in this article are solely those of the authors and do not necessarily represent those of their affiliated organizations, or those of the publisher, the editors and the reviewers. Any product that may be evaluated in this article, or claim that may be made by its manufacturer, is not guaranteed or endorsed by the publisher.
